# The Scandinavian multicenter hemodynamic evaluation of the SJM Regent aortic valve

**DOI:** 10.1186/1749-8090-6-163

**Published:** 2011-12-19

**Authors:** Jon Offstad, Kai Andersen, Per Paulsson, Jesper Andreasson, Ulf Kjellman, Oluf Lundblad, Karl Gunnar Engstrøm, Rune Haaverstad, Jan L Svennevig

**Affiliations:** 1 Oslo University Hospital, Rikshospitalet, PO box 4950, Nydalen, NO 0424 Oslo, Norway; 2 University Hospital, Getingevaegen 4, SE 22185 Lund, Sweden; 3 Blekingesjukhuset, SE 37181 Karlskrona, Sweden; 4 Akademiska sjukhuset, SE 75185 Uppsala, Sweden; 5 University Hospital, SE 70185 Örebro, Sweden; 6 Norrlands Universitetssjukhus, SE 90185, Umeå, Sweden; 7 St. Olavs University Hospital, PO Box 3250, Sluppen, NO 7006 Trondheim, Norway

**Keywords:** Aortic valve replacement, Mechanical heart valve, Cardiac function

## Abstract

**Background:**

112 patients who received small and medium sized St.Jude Regent heart valves (19-25 mm) at 7 Scandinavian centers were studied between January 2003 and February 2005 to obtain non-invasive data regarding the hemodynamic performance at rest and during Dobutamine stress echocardiography (DSE) testing one year after surgery.

**Material and methods:**

46 woman and 66 men, aged 61.8 ± 9.7 (18-75) years, were operated on for aortic regurgitation (17), stenosis (65), or mixed dysfunction (30). Valve sizes were 19 mm (6), 21 mm (33), 23 mm (41), 25 mm (30). Two patients receiving size 27 valves were excluded from the hemodynamic evaluation. Pledgets were used in 100 patients, everted mattress in 66 and simple interrupted sutures in 21. Valve orientation varied and was dependent on the surgeons' choice. 34 patients (30.4%) underwent concomitant coronary artery surgery.

**Results:**

There were two early deaths (1.8%) and three late deaths, one because of pancreatic cancer. Late events during follow-up were: non structural dysfunction (1), bleeding (2), thromboembolism (2). At one year follow up 93% of the patients were in NYHA classes 1-2 versus 47.8% preoperatively.

Dobutamine stress echocardiography (DSE) was performed in a total of 66 and maximal peak stress was reached in 61 patients.

During DSE testing, the following statistically significant changes took place: Heart rate increased by 73.0%, cardiac output by 85.5%, left ventriclular ejection fraction by 19.6%, and maximal mean prosthetic transvalvular gradient by 133.8%, whereas the effective orifice area index did not change.

Left ventricular mass fell during one year from 215 ± 63 to 197 ± 62 g (p < 0.05).

**Conclusion:**

The Dobutamine test induces a substantial stress, well suitable for echocardiographic assessment of prosthesis valve function and can be performed in the majority of the patients. The changes in pressure gradients add to the hemodynamic characteristics of the various valve sizes.

In our patients the St. Jude Regent valve performed satisfactory at rest and under pharmacological stress situation.

## Introduction

The current increase in life expectancy implies increasing need for small and medium size valve replacement since the augmented number of aortic stenosis in the elderly is frequently accompanied by a narrow aortic root. Optimal hemodynamic performance of the valve prosthesis is particularly important in this setting [1,2]. Bileaflet mechanical valves are considered good long-term substitutes, but are still undergoing improvements. As for their assessment, dobutamine stress echocardiography (DSE) has emerged as a measure of the valve hemodynamics adding to the information obtained at rest [3-12]. However, its diagnostic role in this context needs to be further defined.

The purpose of the present multicenter study was to investigate the performance of small and intermediate sizes of the St. Jude Medical Regent aortic valve, with respect to EOA at various stress levels. This prosthesis represents the recent generation of a bileaflet mechanical valve. This would, along with previous results [13], contribute to a large scale assessment. Furthermore, the study investigated the hemodynamic response to dobutamine stress related to the size of normally functioning valves. The responses might supplement their hemodynamic characteristics which need to be assessed before an abnormal response to stress can be defined.

## Material and methods

### Patient Population

A total of 112 patients (46 women and 66 male) undergoing surgery for aortic stenosis (n = 65), aortic regurgitation (n = 17) or both (n = 30) between January 2003 and February 2005 at seven Scandinavian centers were included in the study. Valve sizes were 19 mm (6), 21 mm (33), 23 mm (41), 25 mm (30). Two patients receiving size 27 valves were excluded from the hemodynamic evaluation. Pledgets were used in 100 patients, everted mattress in 66 and simple interrupted sutures in 21. Valve orientation varied and was dependent on the surgeons' choice. 34 patients (30.4%) underwent concomitant coronary artery surgery. Concomitant coronary artery bypass surgery was performed in 34 patients. The age was 61.8 ± 9.7 years (mean-SD), range 18-75 years. Clinically, 99 patients could be evaluated at follow-up one year following surgery: two patients were excluded due to valve size 27 mm, there were two early and three late deaths, two patients developed Hodgkin's disease and there were missing data in four patients.

### Study design

Standard echocardiographic and Doppler examinations were performed preoperatively, at discharge and one year after the operation at each of the seven centers. In addition, DSE was performed at five centers one year after the operation.

### Echocardiographic recordings

Recordings in the parasternal view were obtained of left ventricular size with measurements of intraventricular septum and posterior wall thickness at end-diastole. Left ventricular outflow tract diameter was reported as the dimension just below the prosthetic valve during early systole. Peak and mean velocities in the left ventricular outflow tract was measured from the apical position. Cardiac output was calculated as: CO (l/min) = VTI _LVOT _× CSA × HR, were VTI _LVOT _is the velocity time integral in the left ventricular outflow tract, CSA _(ot) _is the left ventricular outflow tract cross section area, and HR is heart rate.

Additionally flow velocities across the valve were measured. The prosthetic effective orifice area was calculated as: EOA (cm^2^) = CSA _(0t) _× VTI _(ot)_/VTI _ao)_.

EOA _index _: EOA/BSA (cm^2^/m^2^). Performance index was calculated as: PI = EOA/MOA. MOA is manufacture orifice area.

### Dobutamine stress protocol

A standard Dobutamine stress echocardiography (DSE) protocol was performed according to the Mayo clinic protocol (14), starting at a Dobutamine infusion rate of 5 μg/kg/min body weight. The dosage was increased every 5 minutes to 10, 15, 20, 25 and 30 μg/kg/min. Dobutamine stress was discontinued according to established criteria [14]. Blood pressure and 12- lead electrocardiograms were recorded at baseline and at the end of each stage. Images were obtained in the standard parasternal (long and short axis) and apical (2-4-5 chamber and long axis) views. Standard 2 dimension, M mode and Doppler views were recorded at baseline and at the end of the stress protocol.

The Dobutamine stress test was performed whenever available.

### Statistical analysis

Data are presented as mean ± SD. Analysis of variance for repeated measurements were performed with valve size as group factors and time (i.e. before and after the operation or before and during stress). Student t tests for comparison of groups and for comparison of paired data were used whenever appropriate. P < 0.05 was considered significant.

## Consent

Written informed consent to participate in this study was obtained from each patient. A written consent is available for review by the Editot-in-Chif of this journal. The study was approved by the Regional ethical committees.

## Results

There were two early deaths (1.8%). One patient was reoperated for bleeding. The mean follow-up time was 12.3 ± 6.0 months. During follow- up three patients died, one due to a pancreatic carcinoma. Late events during follow-up were: non structural dysfunction (1), bleeding (2), thromboembolism (2).

At follow up, 93 out of 99 patients (94%) were in NYHA classes' 1-2, versus 47.8% preoperatively. Echocardiographic data before surgery, at discharge and one year after operation is presented in Tables 1 and 2. Left ventricular mass was significantly reduced (p < 0.05) after valve replacement, while no significant changes were observed in left ventricular dimensions or ejection fraction (EF). The transvalvular mean pressure gradient was markedly reduced. The only mild residual mean gradients demonstrated a decrease with increasing valve size; 11.4 ± 6.2 mmHg, 9.2 ± 6.6 mmHg, 7.7 ± 3.3 mmHg and 6.0 ± 2.8 mmHg in the sizes of 19, 21, 23 and 25 mm, respectively. Their corresponding EOA indices were 1.0 ± 0.4, 1.0 ± 0.2, 1.0 ± 0.3 and 1.4 ± 0.6 cm^2^/m^2^.

**Table 1:** Left ventricle dimensions, mass and ejection fraction preoperatively, at discharge and after one year

	Preoperatively	Discharge	One year	p
LVDD (mm):	53 ± 9	51 ± 10	50 ± 7	ns
IVD (mm):	13 ± 2	8 ± 1	9 ± 1	ns
LVPW (mm):	11 ± 2	12 ± 2	11 ± 2	ns
Mass (g):	265 ± 85	260 ± 95	214 ± 63	< 0.05
Mass/BSA (g/m^2^):	139 ± 42	137 ± 43	112 ± 30	< 0.05
EF (%):	55.5 ± 10.8	54.3 ± 12.0	58.4 ± 9.5	ns

Mean ± SD, LVDD; left ventricle diastolic dimension,, IVD; intraventricular septum diastolic dimension, LVPW; left ventricle posterior wall dimension, mass; left ventricle mass, Mass/BSA; left ventricle mass index, EF; left ventricle ejection fraction.

**Table 2:** Hemodynamic variables preoperatively, at discharge and after one year

	Preoperatively	Discharge	One year	p
Peak gradient (mmHg):	71.7 ± 32.6	16.8 ± 9.1	5.9 ± 9.0	< 0.01
Mean gradient mmHg):	47.9 ± 22.8	8.7 ± 5.0	8.0 ± 4.8	< 0.01
EOA (cm^2^):	1.6 ± 1.8	2.0 ± 0.7	2.0 ± 0.7	ns
EOA index (cm^2^/m^2^):	0.8 ± 1.0	1.1 ± 0.4	1.1 ± 0.3	ns
Performance index:	0.3 ± 0.2	0.6 ± 0.2	0.6 ± 0.2	< 0.01
CO (l/min):	5.9 ± 1.6	5.8 ± 2.0	5.5 ± 1.4	< 001
CI (l/min/m^2^):	3.1 ± 0.8	3.1 ± 1.0	2.9 ± 0.6	0.01
HR (beat/min):	70 ± 11	81 ± 13	65 ± 13	< 0.01

EOA: effective orifice area, EOA index; effective orifice area index, CO; cardiac output, CI; cardiac index, HR; heart rate.

DSE was performed in 66 patients. There were no serious complications. Maximal peak stress was reached in 61 patients (93%). The test had to be stopped before peak stress was reached in five patients, due to arrhythmia, chest pain or decrease in blood pressure.

The data obtained during DSE are presented in tables 3 and 4. During DSE heart rate increased on average by 73%, CO by 85.5% and the mean transvalvular gradient by 134%, whereas the EOA did not change significantly.

**Table 3:** Hemodynamic variables at rest and during maximal stress (n = 61)

	Rest	Peak stress	p
Heart rate (beat/min):	63 ± 11	109 ± 21	< 0.001
CO (l/min):	5.5 ± 1.4	10.2 ± 3.2	< 0.001
Peak gradient (mmHg):	15.9 ± 9.2	38.3 ± 19.8	< 0.001
Mean gradient mmHg):	8.0 ± 4.8	18.7 ± 10.0	< 0.001
EOA (cm^2^):	2.0 ± 0.7	2.2 ± 0.8	ns
EOA index (cm^2^/m^2^):	1.1 ± 0.4	1.2 ± 0.4	ns
Performance index:	0.55 ± 0.16	0.59 ± 0.20	ns
Ejection fraction (%):	57.8 ± 10.5	69.1 ± 8.6	< 0.001

**Table 4:** Mean pressure gradient, effective orifice area (EOA) and effective orifice area index (EOA index) at rest and during peak Dobutamine stress related to valve area

Valve size:	19	21	23	25	
(n):	(4)	(18)	(23)	(16)	
Mean pressure (mmHg):					p:
At rest:	11.4 ± 6.2	9.2 ± 6.6	7.7 ± 3.3	6.0 ± 2.8	n.s
Peak stress:	27.3 ± 22.0	21.1 ± 10.0	16.9 ± 8.5	16.7 ± 5.8	n.s
EOA (cm^2^):					
At rest:	1.6 ± 0.6	1.8 ± 0.4	1.8 ± 0.5	2.8 ± 1.0	< 0. 001
Peak stress:	2.2 ± 1.5	1.9 ± 0.4	1.8 ± 0.5	2.7 ± 0.7	< 0.02
EOA index (cm^2^/m^2^):					
At rest:	1.0 ± 0.4	1.0 ± 0.2	1.0 ± 0.3	1.4 ± 0.6	n.s.
Peak stress:	1.3 ± 0.9	1.0 ± 0.4	1.2 ± 0.4	1.3 ± 0.4	n.s.

The gradient at peak stress varied according to valve size whereas EOA index did not.

## Discussion

Hemodynamic properties of small sized aortic prostheses have been questioned, due to the possibility of various degrees of hemodynamic functional obstruction [1,2]. Valve prostheses may demonstrate acceptable hemodynamic performance at rest. Additionally, a rise in stroke volume during exercise may increase the pressure drop across the valve and unmask suboptimal valvular function. The hemodynamic performance of aortic valve replacement should therefore be evaluated under various cardiac flow conditions. In this multicenter study hemodynamic evaluation of small mechanical prostheses in the aortic position was performed by transthoracic echocardiography at rest and during DSE.

Stress echocardiography is the combination of 2D echocardiography with a physical, pharmacological or electrical stress [15,16]. Stress echocardiography has been regarded as an important tool in the field of noninvasive diagnosis of coronary artery disease [17,18] and in patients with suspected severe aortic stenosis with low aortic gradients secondary to low cardiac output [19,20].

DSE has additionally been used to examine patients with aortic valve replacement [3-12], mitral valve replacement [20] and double valve replacement. In patients undergoing aortic valve replacement, DSE is an accurate, safe and readily available method to evaluate prostheses hemodynamics [3-12] and to monitor the expected left ventricular hypertrophy regression [12].

The present study demonstrates that DSE can be safely performed in patients one year following heart valve replacement. We used a standard Mayo protocol [14], starting Dobutamine infusion at 5 μg/kg/min. In some previous studies, Dobutamine has been administered to a maximum dose of 20 μg/kg/min [3,4,6,7]. In most of the recent studies a maximum of 40 μg/kg/min was most often used. Co-administration with atropine [15] has not been used in the studies evaluating prosthesis hemodynamics.

In the tested patients heart rate increased by 73% and cardiac output by 85% during DSE. An increase in heart rate from 50-80% and cardiac output from 74-120% have been reported in five studies using the early low dose infusion (20 μg/kg/min) [3,4,6,7]. In three other studies [10-12] using the high dose (40 μg/kg/min), heart rate increased from 69-90% and cardiac output increased from 88 - 110%. As the increase in transvalvular gradients is dependent of flow, the differences in test conditions make comparison of data difficult.

In this study the hemodynamic characteristics of the St. Jude Regent bileaflet prostheses were examined in 66 patients with valve sizes from 19-25 mm. As expected mean transvalvular gradients at rest and during stress decreased with increasing valve size. In the 21 mm sized prostheses mean pressure gradient increased from 9.2 ± 6.6 mmHg to 21.1 ± 10.0 mmHg. These values are associated with a relatively mild transprosthetic gradient at rest and during peak stress. A few studies have previously compared various valve types based on stress tests. Slightly lower pressure gradients and slightly smaller EOA during stress, have been reported by Izzat et al in two studies in 1995 and 1996 [3,4]. These studies included 9 and 10 patients and were performed using bileaflet mechanical valves (Carbo Medics 19-21 mm valves and St. Jude 21 mm). Contrary, a few studies using DSE in 21 mm sized prostheses have documented higher pressure gradients. Kadir et al [8] using the Sorin 21 mm valve, documented that mean transvalvular gradient increased from 15.6 ± 5.3 to 35.4 ± 11.9 mmHg during DSE. In another study [6] using Medtronic Intact aortic bioprotheses, mean transvalvular gradient increased from 19.1 ± 5.1 to 33.2 ± 7.7 mmHg.

In our study the aortic valve area (EOA) remained relatively unchanged despite a significant increase in transprothetic gradients. This finding is in line with the majority of other studies [3,4,6,9,12], and is possibly explained because mechanical components are less prone to accompany larger stroke volumes by increasing the EOA.

An EOAI greater than 0.85-0.9 cm2/m2 has been suggested to minimize the effects of transprosthetic gradient and patient-to-prosthesis mismatch [1,21]. In our study the values for EOAI were above this limit in all sizes both at rest and during peak stress, indicating a favorable hemodynamic performance.

To our knowledge this study is one among the largest using DSE in the postoperative assessment patients following aortic valve replaement. In our experience DSE can be performed safely in all patients and maximum stress can be achieved in the majority of patients. Our study demonstrates excellent hemodynamic results for the St. Jude Medical Regent valve, even at small size, and a significant reduction in the left ventricular mass after one year.

## Conclusion

The Dobutamine test induces a substantial stress, well suitable for echocardiographic assessment of prosthesis valve function and can be performed in the majority of the patients. The changes in pressure gradients add to the hemodynamic characteristics of the various valve sizes. In our patients the St. Jude Regent valve performed satisfactory at rest and under pharmacological stress situation.

## Competing interests

The authors declare that they have no competing interests.

## Authors' contributions

KA and JL. Svennevig were responsible for the study design. The final protocol was agreed on in a censensus meeting. All authors were involved in patients inclusion, treatment, follow-up and collection of data. All authors have read and approved the final manuscript.
